# Circulating microRNA Panel as a Potential Novel Biomarker for Oral Squamous Cell Carcinoma Diagnosis

**DOI:** 10.3390/cancers13030449

**Published:** 2021-01-25

**Authors:** Kodai Nakamura, Naomi Hiyake, Tomofumi Hamada, Seiya Yokoyama, Kazuki Mori, Kouta Yamashiro, Mahiro Beppu, Yasuaki Sagara, Yoshiaki Sagara, Tsuyoshi Sugiura

**Affiliations:** 1 Department of Maxillofacial Diagnostic and Surgical Science, Field of Oral and Maxillofacial Rehabilitation, Graduate School of Medical and Dental Science, Kagoshima University, Kagoshima 890-8544, Japan; pero-pulse-4.14@true.ocn.ne.jp (K.N.); k4639792@kadai.jp (K.M.); k5124979@kadai.jp (K.Y.); mbeppu@dent.kagoshima-u.ac.jp (M.B.); 2 Division of Maxillofacial Diagnostic and Surgical Sciences, Department of Oral and Maxillofacial Surgery, Graduate School of Dental Science, Kyushu University, Fukuoka 812-8582, Japan; hiyake@dent.kyushu-u.ac.jp; 3 Department of Oral & Maxillofacial Surgery, Hakuaikai Medical Cooperation Sagara Hospital, Kagoshima 892-0833, Japan; 4 Department of Pathology, Kagoshima University Graduate School of Medical and Dental Sciences, Kagoshima University, Kagoshima 890-8544, Japan; yokoyama@m3.kufm.kagoshima-u.ac.jp; 5 Department of Breast Surgical Oncology, Hakuaikai Medical Cooperation Sagara Hospital, Kagoshima 892-0833, Japan; yasuaki@sagara.or.jp; 6 Department of Radiology, Hakuaikai Medical Cooperation, Sagara Hospital Kagoshima, Kagoshima 892-0833, Japan; y.sagara@sagara.or.jp

**Keywords:** oral cancer, oral squamous cell carcinoma, microRNA, biomarker, tumor marker

## Abstract

**Simple Summary:**

Although early detection of oral squamous cell carcinoma (OSCC) is considered vital, classical biomarkers have shown poor sensitivity and specificity for early detection and monitoring of OSCC. Therefore, identification of reliable and sensitive biomarkers allowing for early detection and monitoring of OSCC is of the utmost importance. In this study, we successfully identified significantly upregulated or downregulated microRNAs in OSCC patients, and reported that a combination of six microRNAs could distinguish between OSCC and the control group with a higher degree of accuracy. Furthermore, compared with serum squamous cell carcinoma (SCC) antigen, the miRNA panel reflected the presence of OSCC accurately. The present results suggest that the combined microRNA panel based on serum microRNA levels shows potential as a novel diagnostic biomarker of OSCC.

**Abstract:**

A lack of reliable biomarkers for oral squamous cell carcinoma (OSCC) poses a major clinical issue. The sensitivity and specificity of classical serum tumor markers, such as the squamous cell carcinoma antigen (SCC-Ag), are quite poor, especially for early detection. This study aimed to identify specific serum miRNAs potentially serving as OSCC biomarkers. The expression levels of candidate miRNAs in serum samples from 40 OSCC patients and 40 healthy controls were quantitatively analyzed via microarray and reverse transcription PCR (RT-PCR) analyses. To enhance the accuracy of detection, we used Fisher’s linear discriminant analysis to establish a diagnostic model that incorporated a combination of selected miRNAs. Consequently, miR-19a and miR-20a were significantly upregulated in the patient group (*p* = 0.014 and 0.036, respectively), whereas miR-5100 was downregulated (*p* = 0.001). We found that a combination of six miRNAs (miR-24, miR-20a, miR-122, miR-150, miR-4419a, and miR-5100) could distinguish between OSCC and the control group with a higher degree of accuracy (Area Under the Curve, AUC: 0.844, sensitivity: 55%, and specificity: 92.5%). Furthermore, compared to serum SCC antigen, the 6-miRNA panel could accurately detect the presence of OSCC. The present specific miRNAs panel may serve as a novel candidate biomarker of oral cancer.

## 1. Introduction

Oral squamous cell carcinoma (OSCC) is one of the most common cancers worldwide, with the sixth highest incidence among malignant tumors [1]. Early detection of OSCC is vital because the overall survival rate (OS) of patients with stage I and II OSCC is more than 80%, whereas that of stage III and IV patients decreases to approximately 60%. Despite the introduction of new molecular-targeted treatments during the previous decade, the OS rate remains largely unchanged, owing to difficulties in treating advanced OSCC, especially lymph node metastasis and recurrence. Classical biomarkers, such as the squamous cell carcinoma antigen (SCC-Ag) [2,3,4,5], or the carcinoembryonic antigen (CEA) [6,7], have been used to monitor OSCC. However, the sensitivity and specificity of these antigens are quite poor, especially for early detection. Although it is relatively easy to diagnose oral carcinoma through visual examination in the clinic, it is difficult to identify oral cancer during mass screening or during postoperative oral cancer monitoring. Therefore, identification of reliable and sensitive biomarkers for the detection or monitoring of OSCC is of urgent importance.

MicroRNAs (miRNAs) are small noncoding RNAs ranging 18–22 nucleotides in length. Reportedly, miRNAs inhibit mRNA translation by binding to the 3′-untranslated region of the target mRNA [8]. Approximately one-third of human genes may be regulated by miRNAs, indicating that miNRAs may play an important role in cellular regulation. Furthermore, studies have indicated that miRNAs may regulate oncogenes or serve as tumor suppressors in numerous types of human cancers, as shown by the finding that approximately 50% of the human miRNAs are located in cancer-associated genomic regions or in fragile sites [9]. In various types of cancers, altered miRNA profiles in cancer cells are associated with malignant characteristics, where miRNA expression profiles are tumor type-specific [10]. Several types of miRNA alterations have been reported in OSCC tissues and cell lines [11]. Some miRNA profiles of OSCC are associated with the metastasis [11].

Moreover, miRNAs are stable and detectable in tissues and in body fluids, such as plasma and serum, owing to protection from RNases [12,13]. Based on the activity and stability of circulating miRNAs in body fluids, these have been utilized as noninvasive diagnostic and prognostic biomarkers of various malignant tumors [14,15]. Similarly, studies screening circulating miRNA biomarkers of OSCC are underway [16,17,18]. However, to date, candidate miRNAs, whose diagnostic performance is suitable in clinical examinations for detection of OSCC, have not been identified. Therefore, this study aimed to investigate miRNA expression to establish a method with a high diagnostic capability for OSCC detection. The present results suggest that a method combining serum microRNAs as a diagnostic panel may be useful for the detection of oral cancer.

## 2. Results

### 2.1. Clinicopathological Characteristics of Study Participants

Clinicopathological characteristics of 40 OSCC patients and 40 healthy controls are listed (Table 1). The rate of pathological regional lymph node metastasis at the time of surgery was 30.0%. No significant differences were observed between the age and sex proportion of the OSCC group and the control group (Table 1).

### 2.2. Comprehensive Analysis of Serum miRNA via Microarray Analysis

**Figure 1:**
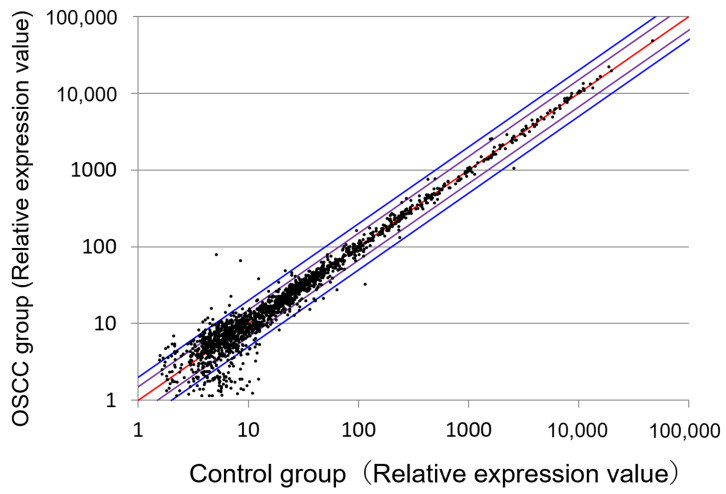
Comprehensive analysis of serum miRNA using a microarray. Microarray analysis detected 48 upregulated and 40 downregulated oral squamous cell carcinoma (OSCC)-specific miRNAs in patients, compared to healthy controls.

RNA profiles in the serum pools of 10 OSCC patients were compared with those of 10 healthy controls via miRNA microarray analysis to identify candidate miRNAs as potential OSCC markers (Figure 1).

**Figure 2:**
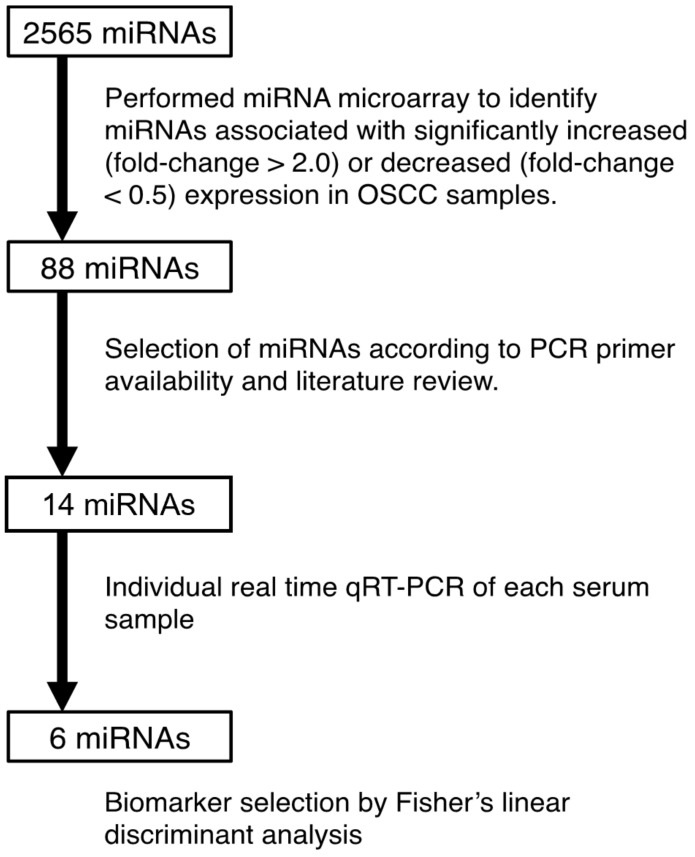
Flow diagram for detection of candidate microRNAs.

Of the 2565 miRNAs in the OSCC pooled serum samples that were scanned, 48 were upregulated (>2-fold) and 40 were downregulated (<0.5-fold) (Figure 2, Table S1). Of the 88 miRNAs with a large difference in expression level between the OSCC and control groups, candidate miRNAs were selected when (1) they had a significant signal intensity (25>) after global normalization (average signal intensity of the chip was adjusted to 25), and (2) the primer for the TaqMan^®^ Assay (Applied Biosystems, Foster City, CA, USA) was commercially available. From these, we selected 14 miRNAs for the validation experiment on the basis of primer availability and literature review (Table S2).

### 2.3. Validation of Candidate miRNAs via Real Time RT-PCR

**Figure 3:**
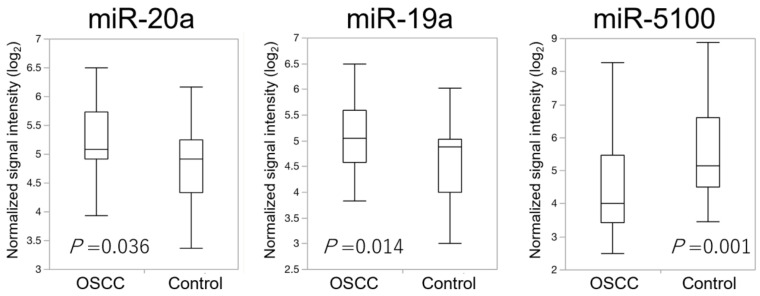
Comparison of normalized signal intensities of three microRNAs in oral squamous cell carcinoma (OSCC) patients and the control group. Signal intensities of three microRNAs, miR-20a, miR-19a, and miR-5100, were significantly different (Wilcoxon test, *p* < 0.05). Relative expression levels were significantly increased in miR-19a and miR-20a of OSCC patients, whereas miR-5100 of the control group showed a higher intensity.

We quantitatively evaluated expression levels of the 14 selected miRNAs in the sera of 40 OSCC patients and 40 healthy controls. miR-19a and 20a in the sera of OSCC patients were significantly upregulated compared to those in the sera of healthy controls (*p* = 0.014 and *p* = 0.036, respectively, Figure 3). By contrast, miR-5100 was significantly downregulated in the sera of OSCC patients when compared to that in the sera of healthy controls, which contradicted microarray results (*p* = 0.001, Figure 3). No significant difference between OSCC and control groups was observed regarding the expression levels of 11 of the 14 miRNAs evaluated in this study (Figure S1).

### 2.4. Diagnostic Performance of OSCC Detection via Single microRNA Analysis

**Figure 4:**
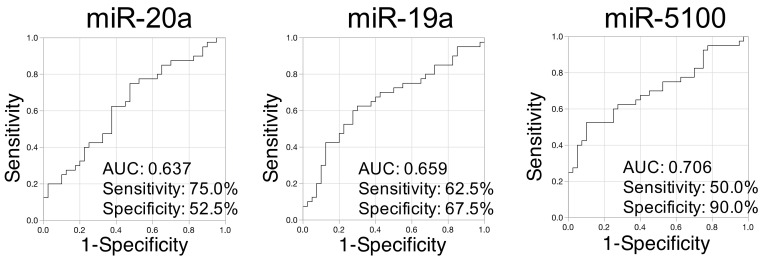
Receiver operating characteristic (ROC) analyses for miR-20a, miR-19a, and miR-5100 indicating a relatively higher area under the curve (AUC).

To obtain an appropriate cutoff value for the signal intensity level of each miRNA, receiver operating characteristic (ROC) curves were generated for the 14 miRNAs and AUC values that were calculated (Figure 4). The AUC, sensitivity, specificity, positive predictive value (PPV), and negative predictive value (NPV) for OSCC detection and results of the Fisher’s exact test are listed (Table 2). Based on the cutoff value, the Fisher’s exact test indicated a significant difference in the signal intensities between the OSCC and control groups for eight ((miR-19a, miR-19b, miR-20a, miR-423, miR-122, miR-144, miR-183, and miR-5100) of the 14 miRNAs (Table 2). In particular, the AUC for miR-5100 was >0.7, suggesting that it was adequate for cancer detection (Figure 4, Table 2). ROC curves and AUC values for the other 11 miRNAs are listed in Figure S2.

### 2.5. Diagnostic Performance of OSCC Detection via the 6-miRNA Panel

For more precise detection, we performed combined analysis using the Fisher’s linear discriminant analysis. Statistical analysis identified a combination of six miRNAs (miR-24, miR-20a, miR-122, miR-150, miR-4419a, and miR-5100), for which the diagnostic index (miRNA index) was as follows (1).

miRNA index = −0.31567 + (0.09049 * miR-24) + (0.21063 * miR-20a) − (0.04705 * miR-122) − (0.08418 * miR-150) + (0.14492 * miR-4419a) − (0.22962 * miR-5100)(1)

**Figure 5:**
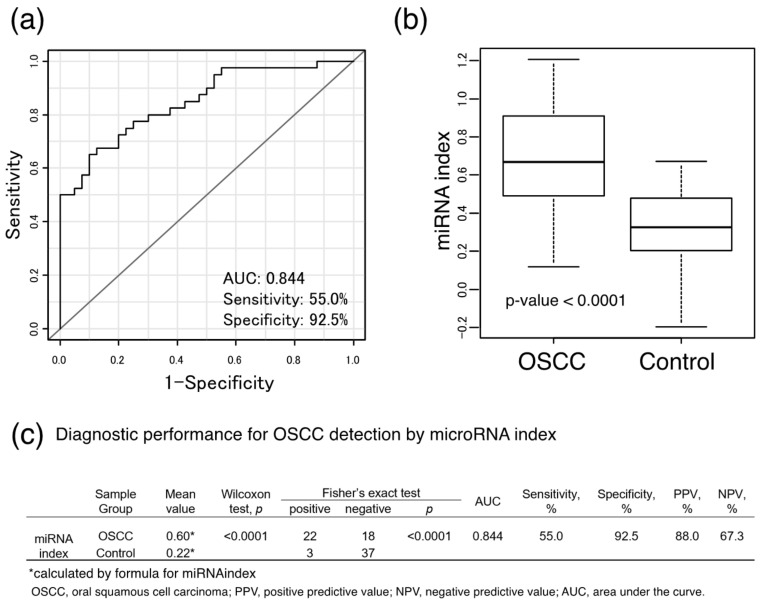
The diagnostic performance of the models was investigated using a combination of miRNAs. (**a**) Using the Fisher’s linear discriminant analysis, the diagnostic index (named as miRNA index) was expressed as Equation (1). (**b**) The level of the miRNA index Q-TPJ, Chen Z. in other references in the listed 10 authors were listed prior to the additional meaning. NPV is also appropriate here, which was demonstrated to be significantly higher in the oral squamous cell carcinoma (OSCC) patient group than that in the control group. (*p* < 0.0001, Wilcoxon test). (**c**) Diagnostic performance for OSCC detection by the miRNA index using the cutoff value.

The diagnostic performance of this panel revealed a high degree of accuracy (AUC: 0.844, sensitivity: 55%, specificity: 92.5%, positive predictive value (PPV): 88.0%, and negative predictive value (NPV): 67.3%), (Table 2, Figure 5). This 6-miRNA panel predicted the presence of OSCC with statistical significance by distinguishing it from the control group. The combined model demonstrated a higher diagnostic power than that of a single microRNA.

### 2.6. The Diagnostic Performance of 6-miRNA Panel Compared with a Squamous Cell Carcinoma Antigen

**Figure 6:**
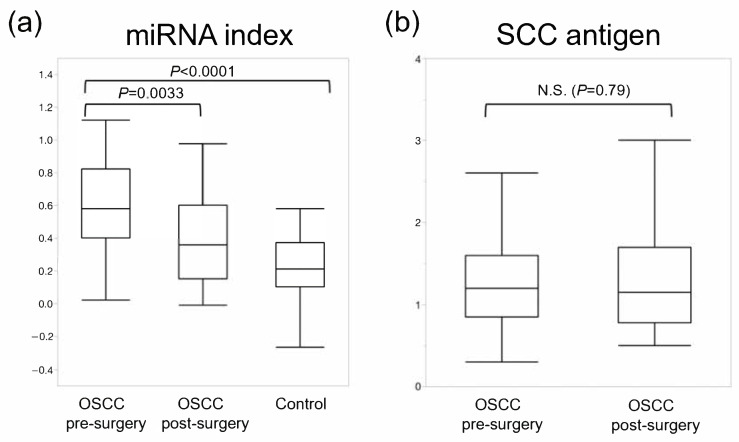
The diagnostic performance of the combination of miRNAs compared with that of a conventional serum tumor marker. (**a**) The miRNA index level of the OSCC group (OSCC pre-surgery) was significantly higher than that of the post-surgery OSCC group and the control group (*p* < 0.0001 and *p* = 0.0033, respectively, Wilcoxon test). (**b**) The serum squamous cell carcinoma (SCC) antigen level showed no significant difference between pre-surgery and post-surgery OSCC groups (*p* = 0.79, Wilcoxon test).

Lastly, we compared the diagnostic performance of the miRNA panel with that of a classical serum biomarker. The levels of squamous cell carcinoma antigen (SCC-Ag) in the sera of OSCC patients were quantified at pre-surgery and post-surgery points. The miRNA index level in the post-surgery OSCC group (OSCC post-surgery) was significantly lower than that of the pre-surgery OSCC group (*p* < 0.003, Wilcoxon test) (Figure 6 and Table 3) and approximated to that of the control group, suggesting that the 6-miRNA panel reflected the presence of OSCC. However, serum SCC antigen levels were not significantly different between pre-surgery and post-surgery in the OSCC group (Figure 6 and Table 3). The sensitivity of the serum SCC antigen for OSCC detection was 8.1% (3/37).

## 3. Discussion

This study aimed to identify miRNAs potentially serving as diagnostic biomarkers of OSCC. Previous studies reported that the expression pattern of miRNAs in saliva was altered during the early stages of OSCC development, indicating its utility for early detection of OSCC or precancerous lesions [20,21,22,23,24,25]. Since the current study was designed to improve the detection of OSCC even in postoperative metastases, we selected circulating miRNAs. Our initial challenge was to determine the origin of circulating miRNAs, because, according to some reports, miRNAs may be isolated from serum, plasma, or purified exosomes obtained from blood. Exosomes contain a wide array of biological information, such as those pertaining to genes and proteins as well as miRNAs. Furthermore, miR-1246, miR-4644, miR-3976, and miR-4306, obtained from purified exosomes in the serum, have been identified as diagnostic biomarkers of pancreatic cancer [20]. Some studies have reported the stability of exosomes during handling as well as during freeze and thaw cycles [20,26,27].

Our preliminary attempts to purify exosomes from blood samples were not successful as the purity and quantity of exosomes were not reproducible. Furthermore, this procedure is costly and time-consuming. Another approach is the detection of circulating miRNA for clinical use. This method is sensitive, specific, rapid, and inexpensive. Therefore, screening of circulating miRNAs in the serum was considered. Screening was performed using pooled serum samples from 10 OSCC patients and 10 healthy controls, using an miRNA microarray platform, as per the methods described previously [28].

Classic serum tumor markers such as squamous cell carcinoma antigen (SCC-Ag), carcinoembryonic antigen (CEA), and carbohydrate antigen 19-9 (Ca19-9) have been long used for detection of OSCC. However, reports regarding the accuracy of these markers in diagnosis of OSCC are lacking. SCC-Ag, detected in advanced stage IV patients, is useful as a prognostic marker to monitor the progression of OSCC [2,29]. However, SCC-Ag is used as a monitoring marker of cervical squamous cell carcinoma. Studies have revealed that it is not as sensitive (AUC 0.713, sensitivity, 0.612, specificity, 0.700) as the new serum miRNA markers [30]. The current study indicated that the expression levels of three specific miRNAs (miR19a, 20a, 5100) were significantly altered in the OSCC group, even though these alterations did not indicate a significant increase or decrease in tumor progression (stage classification, data not shown). Hence, the diagnostic value of our target miRNAs, which is also superior to that of SCC-Ag for cervical SCC, may be considered acceptable, and these miRNAs may be considered OSCC diagnostic markers. Numerous studies have attempted to identify non-miRNA serum OSCC markers. To date, Gas6 (sensitivity of 63% with a specificity of 92%) [31], galectin-1 (sensitivity of 85.71% with a specificity of 84.38%), and galectin-3 (sensitivity of 83.93% with a specificity of 84.38%) [32] have been reported. The sensitivity and specificity of our miRNA markers were comparable to those of the previously mentioned markers.

This study reveals a significant difference between OSCC and control groups with regard to the expression levels of miR-19a, miR-20a, and miR-5100. Our results show that miR-19a and miR-20a levels in OSCC patients were significantly increased when compared with those in the serum of healthy controls. By contrast, miR-5100 expression in the sera of OSCC patients was significantly decreased. Moreover, miR-19a is an oncomiR, the expression level of which was reportedly increased in patients with numerous malignant cancers, such as breast cancer [33] and lung cancer [34]. It has been suggested that, in osteosarcoma, miR-19a inhibits the JAK2/STAT3 signaling pathway, activates the mitochondrial apoptotic pathway, and promotes the expression of apoptosis-related proteins [35]. Additionally, miR-19a-3p levels, which reportedly impacted the PTEN/PI3K/AKT pathway, were evidently increased in gastric cancer tissues and cells [36]. Li et al. reported that, in oral cancer, TGFBR3 (transforming growth factor type III receptor) is a direct target of miR-19a, which also promotes epithelial-to-mesenchymal transition and migration of tongue squamous cell carcinoma cells in vitro [37]. This study shows that miR-19a serves as a marker of OSCC, thus, corroborating previous findings. Therefore, the expression levels of these miR-19a targets should be investigated in future studies.

Reportedly, miR-20a is an oncomiR that participates in cell proliferation and cancer progression [38]. Meta-analysis has demonstrated that miR-20a levels in tumor samples of colorectal cancer patients are elevated, supporting the hypothesis that miR-20a is a sensitive diagnostic tool in colorectal cancer [38]. Furthermore, miR-20a has been reported to be highly expressed in esophageal squamous cell carcinoma [39]. Geng et al. have reported that five plasma miRNAs, including miR-20a, may be used as promising biomarkers in the early screening of non-small cell lung cancer [40]. miR-20a reportedly inhibits cell migration in oral cancer and, thus, may be used as a prognostic marker for this disease [41], suggesting that miR-20 and miR-19a, may function as oncogenic miRNAs (onco-miR) in oral squamous cell carcinoma. Numerous tumor-related genes, such as *HMGA2* [42], *RB1CC1/FIP200* [43], *SRCIN1* [44], *STAT3* [45], and *PTEN* [46], are targeted by miR-20a. Therefore, miR-20a-targeted therapy may strongly impact oral cancer.

This study shows that miR-5100 was significantly downregulated in OSCC patients. Zhang et al. have suggested that miR-5100 serves as a tumor suppressor in gastric cancer cells [47]. Similarly, Chijiiwa et al. have reported that miR-5100 exerts an inhibitory effect on the occurrence and metastasis of pancreatic cancer [48]. These reports are consistent with our results. However, several studies have reported that miR-5100 is upregulated in lung cancer [49], colon cancer [50], and oral squamous cell carcinoma [51]. These inconsistencies suggest that miR-5100 may act as an oncomiR and/or anti-oncomiR depending on the tissue/cancer type and the period of carcinogenesis. In this regard, a few reports pertaining to the role of miR-5100 are available, with particular reference to the field of oral cancer. Further studies on different types of cancers may be required in order to clarify its biological function, including target genes. The reason for this inconsistency may be the methodology of microRNA detection and the size of samples used in our study. One of the limitations of this study is that cohort sizes of OSCC patients and healthy subjects were small. Further studies using a larger patient population may be warranted to address these issues. To date, among the microRNAs selected in this study, only a few in vitro experiments were conducted using an oral cancer cell line. The in vitro research studies that focus on biological functions, targets, and signals of microRNA using silencing/overexpressing or antibody-based methods is essential for understanding for biology of oral cancer in the future. Furthermore, bioinformatic approaches of microRNA study have the potential to predict the target of drug therapy for oral cancer in the future.

## 4. Materials and Methods

### 4.1. Study Design and Subjects

This study included 40 healthy individuals and 40 patients who were newly diagnosed with OSCC at the Department of Oral Surgery, Kagoshima University Hospital (Kagoshima, Japan) between 2016 and 2018. Clinical data from each case, including age, sex, location, tumor size, and nodal status, were obtained from patient files. Staging was performed for all patients in accordance with the criteria of the Union for International Cancer Control tumor-node-metastasis (TNM) classification of malignant tumors. Forty healthy individuals were recruited among patients undergoing routine health screening at Kagoshima University Hospital (Kagoshima, Japan), and presented no pathognomonic signs.

### 4.2. Ethics, Consent, and Permission

The protocol for this research project was approved (code: 28-159; date: 5 September 2016) by the Ethics Committee of Kagoshima University. Written informed consent was obtained from all patients and normal individuals before the commencement of the study. This study was performed in accordance with the tenets of the 1975 Declaration of Helsinki.

### 4.3. Serum Sampling

All blood samples from OSCC patients were collected prior to treatment (14 days before surgery). Post-operative samples were also obtained from 40 patients six months following surgical treatment. Whole blood samples (10 mL) were collected in serum separator tubes from all participants. For complete clotting, the tubes were allowed to stand at room temperature for 30 min, which was followed by centrifugation for 10 min at 1900× *g* at 4 °C. The upper serum fraction was recovered and additional centrifugation for 10 min at 1600× *g* at 4 °C was performed to eliminate cell debris. Supernatants used as serum samples were stored at −80 °C until analysis.

### 4.4. RNA Isolation

Total RNA (including small RNA) was isolated from 200-µL serum samples using the miRNeasy Serum/Plasma Kit (Qiagen, Chatsworth, CA, USA) and carrier RNA (MS2 RNA, Roche Diagnostics, Mannheim, Germany) following the manufacturer’s instructions. Isolated RNA was directly used for miRNA microarray analysis and real-time reverse transcription-PCR (RT-PCR).

### 4.5. miRNA Microarray

First, miRNA microarray analysis was performed to identify differentially expressed miRNAs in a serum sample pooled from 10 randomly selected OSCC patients and 10 healthy controls. Serum pool RNA was isolated as described in the “RNA isolation” section and labeled using the 3D-Gene miRNA labeling kit (Tray Industries, Kamakura, Japan) in accordance with the manufacturer’s instructions. Labeled RNAs were hybridized onto the 3D-gene miRNA array platform (2565 miRNAs, ver.21, Toray, Kamakura, Japan). The annotation and oligonucleotide sequences of the probes conformed to the miRBase miRNA database (http://microrna.sanger.ac.uk/sequences/). Following stringent washes, fluorescent signals were scanned using the 3D-Gene Scanner (Toray Industries, Kamakura, Japan) and analyzed using the 3D-Gene Extraction software (Toray Industries, Kamakura, Japan).

The raw data of each spot were normalized by substituting the mean intensity of the background signal determined from the signal intensities of all blank spots with a 95% confidence interval. Spots with signal intensities greater than 2 standard deviation values of the background signal intensity were considered valid. The relative expression level of a particular miRNA was determined by comparing the signal intensities of valid spots throughout the microarray experiments. Normalized data were globally normalized per array by adjusting the median of the signal intensity to 25. Relative hybridization intensities and background hybridization values were determined. Significant changes were defined as >2.0-fold or <0.5-fold by comparing the results of OSCC patients to those of healthy controls.

### 4.6. Quantitative Real Time RT-PCR

Expression levels of 15 selected miRNAs in each serum sample obtained from 40 OSCC patients and 40 healthy controls were measured using quantitative real time RT-PCR using the TaqMan^®^ MicroRNA Assays (Applied Biosystems, Foster City, CA, USA) and TaqMan^®^ Universal PCR Master Mix II (Applied Biosystems, Foster City, CA, USA) in accordance with the manufacturer’s instructions. The probes for miRNAs were purchased from Applied Biosystems (Foster City, CA, USA). PCR cycling conditions were as follows: 10 min at 95 °C for 1 cycle, followed by 45 cycles at 95 °C for 15 s, and 60 °C for 60 s. Owing to the lack of an internal control for miRNAs, we selected miR-16 as an endogenous internal control, which showed no change in expression between OSCC patients and control groups in the miRNA microarray. We also analyzed the expression levels of candidate internal control miRNAs using real time RT-PCR. According to preliminary statistical analysis, miR-16 was selected as the stable internal control because of a stable higher expression level in all samples (mean threshold cycle, CT value in all samples = 26.28, Table S3), smaller standard deviation (SD = 1.8, Table S3), normal distribution, and no difference of expression level between two groups (CT value = 26.25 in a cancer group and 26.31 in a control group, respectively). In addition, miR-16 is now said to exist stably in blood samples and has been used as control miRNA in previously reported studies [52,53,54]. The expression levels (relative expression level of healthy controls) of target miRNAs in each sample were evaluated using the 2-ddCt method (CT = cycle threshold) as described previously [20], using the following formula.

1. ddCT(target miR) patient = [CT(target miR) patient—CT (miR-16) patient]—dCT healthy control
2. dCT healthy control = Mean value of [CT (target miR) healthy control—CT (miR-16) healthy control]

## 5. Conclusions

In conclusion, we successfully identified differentially expressed microRNAs in OSCC patients. The present results indicate that OSCC can potentially be detected with relatively high sensitivity and specificity on the basis of serum microRNA levels. The panel of specific miRNAs showed potential as a novel biomarker for OSCC.

## Figures and Tables

**Table 1:** Clinicopathological characteristics of study participants.

Variable	OSCC Group (*n* = 40)	Control Group (*n* = 40)	*p*-Value
Age, mean (years)	67.3	63.7	NS (*p* = 0.30)
Sex, *n* (%)			
Male	21 (52.5)	20 (50.0)	NS (*p* = 0.82)
Female	19 (47.5)	20 (50.0)	
Location, *n* (%)			
Tongue	21 (52.5)		
Gingiva	14 (35.0)		
Oral floor	4 (10.0)		
Buccal mucosa	1 (2.5)		
T classification, *n* (%)			
T1	4 (10.0)		
T2	16 (40.0)		
T3	7 (17.5)		
T4	13 (32.5)		
N classification, *n* (%)			
N > 0	12 (30.0)		
N0	28 (70.0)		
M classification, *n* (%)			
M > 0	0 (0.0)		
M0	40 (100.0)		
Stage classification, *n* (%)			
I	3 (7.5)		
II	14 (35.0)		
III	8 (20.0)		
IV	15 (37.5)		
Tumor differentiation, *n* (%)			
Well-differentiated	35 (87.5)		
Moderate	5 (12.5)		
Vascular invasion, *n* (%)			
(+)	14 (35.0)		
(−)	26 (65.0)		
Lymphatic invasion, *n* (%)			
(+)	3 (7.5)		
(−)	37 (92.5)		
Perineural invasion, *n* (%)			
(+)	7 (17.5)		
(−)	33 (82.5)		
Mode of invasion *, *n* (%)			
1	0 (0.0)		
2	1 (2.5)		
3	29 (72.5)		
4C	6 (15.0)		
4D	4 (10.0)		

OSCC, oral squamous cell carcinoma; NS, Not Significant; * Yamamoto-Kohama (YK) Classification [19].

**Table 2:** Diagnostic performance of miRNAs in oral squamous cell carcinoma (OSCC) detection using cutoff values.

microRNA	Sample Group	Mean Value	Wilcoxon Test, *p*	Fisher’s Exact Test			AUC	Sensitivity, %	Specificity, %	PPV, %	NPV, %
				Positive	Negative	*p*					
mir23	OSCC	6.04	0.92	30	10	0.5856	0.494	75.0	17.5	47.6	41.2
	Control	6.17		33	7						
											
mir24	OSCC	4.49	0.89	24	16	0.8176	0.491	60.0	35.0	48.0	46.7
	Control	4.51		26	14						
											
mir423	OSCC	5.48	0.23	5	35	0.0188	0.579	87.5	37.5	75.0	58.3
	Control	5.84		15	25						
											
mir19a	OSCC	5.02	0.014	25	15	0.0133	0.659	62.5	67.5	65.8	64.3
	Control	4.67		13	27						
											
mir19b	OSCC	4.09	0.28	16	24	0.01	0.571	40.0	87.5	76.2	59.3
	Control	3.90		5	35						
											
mir20a	OSCC	5.21	0.036	30	10	0.021	0.637	75.0	52.5	61.2	67.7
	Control	4.88		19	21						
											
mir22	OSCC	8.06	0.22	25	15	0.0784	0.580	62.5	17.5	43.1	31.8
	Control	8.20		33	7						
											
mir122	OSCC	6.93	0.09	31	9	0.0143	0.609	22.5	97.5	55.7	90.0
	Control	8.41		39	1						
											
mir125	OSCC	9.16	0.42	19	21	0.2611	0.553	47.5	37.5	43.2	41.7
	Control	9.45		25	15						
											
mir144	OSCC	8.80	0.18	18	22	0.0307	0.588	45.0	80.0	69.2	59.3
	Control	8.44		8	32						
											
mir183	OSCC	13.09	0.07	22	18	<0.0001	0.616	45.0	95.0	63.3	90.0
	Control	13.35		38	2						
											
mir150	OSCC	7.84	0.48	31	9	0.2247	0.546	22.5	90.0	53.7	69.2
	Control	8.17		36	4						
											
mir4419a	OSCC	9.86	0.49	22	18	0.0576	0.546	45.0	77.5	58.5	66.7
	Control	10.11		31	9						
											
mir5100	OSCC	4.51	0.001	20	20	0.0002	0.706	50.0	90.0	64.3	83.3
	Control	5.60		36	4						
miRNA index	OSCC	0.60*	<0.0001	22	18	<0.0001	0.844	55.0	92.5	88.0	67.3
	Control	0.22*		3	37						

* Caluculated by formula for miRNAindex; OSCC, oral squamous cell carcinoma; AUC, Area Under the Curve; PPV, positive predictive value; NPV, negative predictive value.

**Table 3:** Diagnostic performance of the miRNA index and serum squamous cell carcinoma (SCC) antigen among pre-surgery and post-surgery oral squamous cell carcinoma (OSCC) groups and the control group.

		miRNA Index					Serum SCC Antigen	
		Mean	p *	Fisher’s Exact Test			Mean	p *
				Positive	Negative	p **		
OSCC	pre-surgery	0.595	ref	22	18	ref	1.676	ref
	post-surgery	0.400	0.0033	12	28	0.04	1.503	0.79
Control		0.217	<0.0001	3	37	<0.0001	ND	-

* Wilcoxon test; ** Fisher’s exact test; ref, reference; ND, not determined.

## Data Availability

Data available on request due to restrictions of ethical policy.

## Supplementary Materials

The following are available online at https://www.mdpi.com/2072-6694/13/3/449/s1. Figure S1: Comparison of normalized signal intensity of 11 microRNAs between oral squamous cell carcinoma (OSCC) patients and a control group, Figure S2: ROC analyses for 11 microRNAs, Table S1: Upregulated (>2-fold), and downregulated miRNAs (<0.5-fold) in OSCC patients selected for miRNA microarray analysis, Table S2: List of miRNAs used for RT-PCR validation, Table S3: CT value of each miRNA in all samples.
