# Supplementary material for: Circulating microRNA Panel as a Potential Novel Biomarker for Oral Squamous Cell Carcinoma Diagnosis
Source: Cancers (Basel). 2021 Jan 25;13(3):449. doi: 10.3390/cancers13030449 (PMC7865311; doi:10.3390/cancers13030449)
Supplement: Supplementary file 1 [file cancers-13-00449-s001.pdf]

cancers

MDPI

*Supplementary Files*

# Circulating microRNA Panel as a Potential Novel Biomarker for Oral Squamous Cell Carcinoma Diagnosis

**Kodai Nakamura, Naomi Hiyake, Tomofumi Hamada, Seiya Yokoyama, Kazuki Mori, Kouta Yamashiro, Mahiro Beppu, Yasuaki Sagara, Yoshiaki Sagara and Tsuyoshi Sugiura**

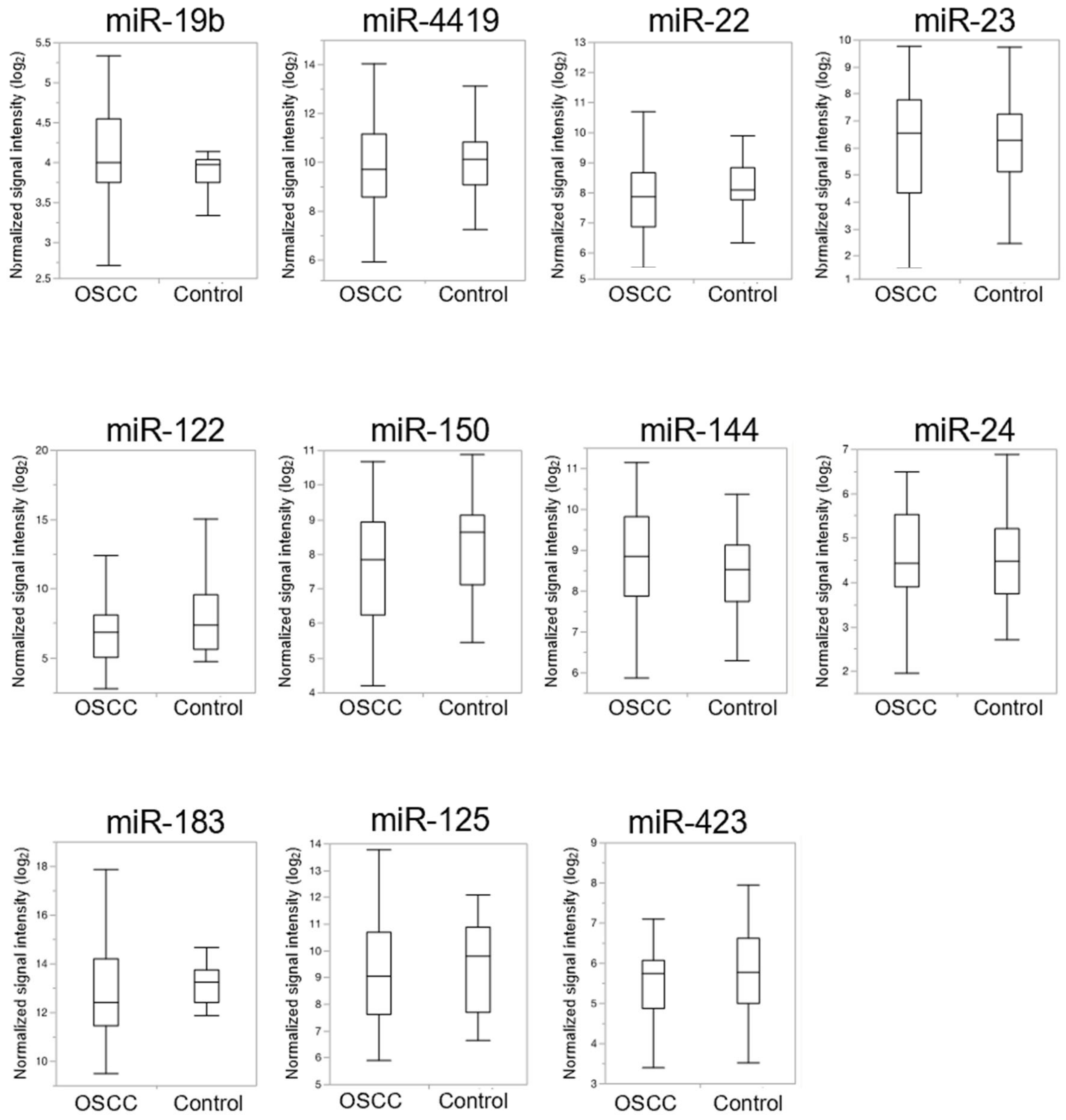


**Figure S1.** Comparison of normalized signal intensity levels of 11 microRNAs of oral squamous cell carcinoma (OSCC) patients and the control group. No significant difference was observed between these microRNAs.

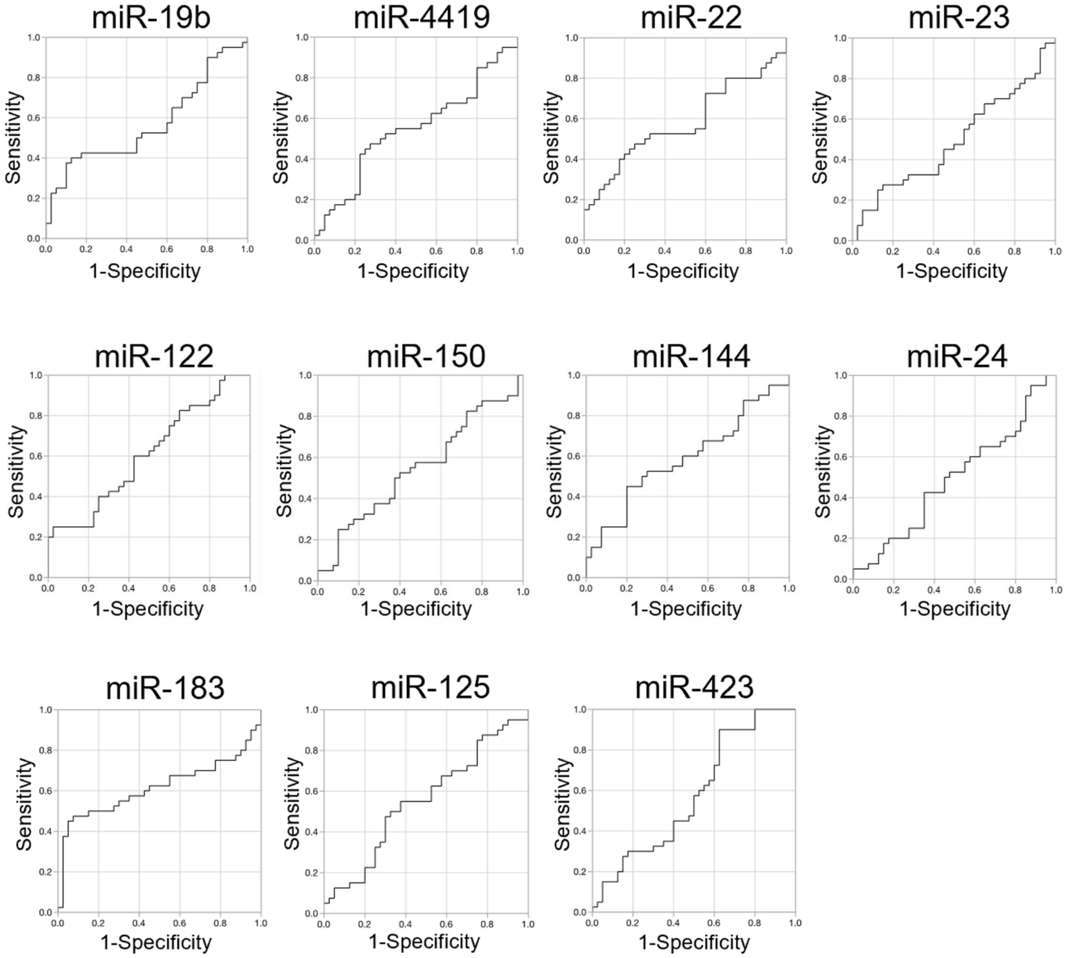


**Figure S2.** Receiver operating characteristic analyses for 11 miRNAs that did not exhibit significant differences between oral squamous cell carcinoma patients and the control group.

**Table S1.** Upregulated (>2-fold) and downregulated (<0.5-fold) miRNAs selected for miRNA microarray analysis from among OSCC patients.

| Upregulated miRNA | Fold Change of OSCC/Control | Downregulated miRNA | Fold Change of OSCC/Control |
|---|---|---|---|
| hsa-miR-144-3p | 10.62 | hsa-miR-4745-3p | 0.36 |
| hsa-miR-19a-3p | 8.27 | hsa-miR-5195-5p | 0.38 |
| hsa-miR-20a-5p | 4.86 | hsa-miR-7153-5p | 0.38 |
| hsa-miR-451a | 4.42 | hsa-miR-5100 | 0.40 |
| hsa-miR-26b-5p | 4.40 | hsa-miR-622 | 0.40 |
| hsa-miR-130a-3p | 4.27 | hsa-miR-6842-3p | 0.40 |
| hsa-miR-18b-5p | 4.24 | hsa-miR-4307 | 0.43 |
| hsa-miR-106b-5p | 4.00 | hsa-miR-7107-3p | 0.44 |
| hsa-miR-19b-3p | 3.99 | hsa-miR-6718-5p | 0.45 |
| hsa-miR-21-5p | 3.94 | hsa-miR-4644 | 0.46 |
| hsa-miR-126-5p | 3.91 | hsa-miR-3666 | 0.47 |
| hsa-miR-423-5p | 3.89 | hsa-miR-1285-5p | 0.47 |
| hsa-miR-29c-3p | 3.85 | hsa-miR-4502 | 0.48 |
| hsa-miR-208a-5p | 3.80 | hsa-miR-4784 | 0.48 |
| hsa-miR-3194-3p | 3.65 | hsa-miR-766-5p | 0.48 |
| hsa-miR-16-5p | 3.40 | hsa-miR-7112-3p | 0.48 |
| hsa-let-7i-5p | 3.36 | hsa-miR-29c-5p | 0.49 |
| hsa-miR-30e-5p | 3.33 | hsa-miR-363-5p | 0.50 |
| hsa-miR-23a-3p | 3.31 | hsa-miR-6864-3p | 0.51 |
| hsa-miR-143-3p | 3.20 | hsa-miR-3976 | 0.51 |
| hsa-miR-363-3p | 3.20 | hsa-miR-6809-5p | 0.51 |
| hsa-miR-324-5p | 3.19 | hsa-miR-6739-3p | 0.52 |
| hsa-miR-525-5p | 3.17 | hsa-miR-7-2-3p | 0.53 |
| hsa-miR-452-5p | 3.15 | hsa-miR-4419a | 0.53 |
| hsa-miR-142-5p | 3.03 | hsa-miR-1266-5p | 0.53 |
| hsa-miR-5093 | 2.91 | hsa-miR-4470 | 0.54 |
| hsa-miR-24-3p | 2.85 | hsa-miR-181a-2-3p | 0.55 |
| hsa-miR-20b-5p | 2.75 | hsa-miR-125b-2-3p | 0.55 |
| hsa-miR-4800-3p | 2.75 | hsa-miR-519e-5p | 0.55 |
| hsa-miR-4289 | 2.75 | hsa-miR-3927-5p | 0.55 |
| hsa-miR-22-3p | 2.74 | hsa-miR-5684 | 0.55 |
| hsa-miR-15a-5p | 2.72 | hsa-miR-550a-5p | 0.55 |
| hsa-miR-3170 | 2.64 | hsa-miR-4717-5p | 0.56 |
| hsa-miR-591 | 2.63 | hsa-miR-150-3p | 0.56 |
| hsa-miR-194-5p | 2.53 | hsa-miR-4317 | 0.56 |
| hsa-miR-122-5p | 2.51 | hsa-miR-4446-5p | 0.56 |
| hsa-miR-151a-3p | 2.49 | hsa-miR-4421 | 0.56 |
| hsa-let-7f-5p | 2.48 | hsa-miR-6074 | 0.57 |
| hsa-miR-8080 | 2.48 | hsa-miR-517a-3p | 0.57 |
| hsa-miR-205-5p | 2.45 | hsa-miR-566 | 0.57 |
| hsa-miR-27a-3p | 2.45 | | |
| hsa-miR-181d-5p | 2.43 | | |
| hsa-miR-93-5p | 2.40 | | |
| hsa-miR-3660 | 2.35 | | |
| hsa-miR-146b-5p | 2.32 | | |
| hsa-miR-7515 | 2.32 | | |
| hsa-miR-126-3p | 2.30 | | |
| hsa-miR-183-5p | 2.30 | | |

**Table S2.** List of miRNAs used for RT-PCR validation.

| **miRNA** |
| --- |
| hsa-miR-144-3p |
| hsa-miR-19a-3p |
| hsa-miR-20a-5p |
| hsa-miR-19b-3p |
| hsa-miR-423-5p |
| hsa-miR-23a-3p |
| hsa-miR-24-3p |
| hsa-miR-22-3p |
| hsa-miR-122-5p |
| hsa-miR-183-5p |
| hsa-miR-5100 |
| hsa-miR-4419a |
| hsa-miR-125b-2-3p |
| hsa-miR-150-3p |

**Table S3.** CT value of each miRNA in all samples (cancer and control group, *n* = 80).

| | CT Value | | | | | |
|---|---|---|---|---|---|---|
| **miRNA** | **Mean** | **Minimum** | **Maximum** | **SD** | **Shapiro-Wilk Test *p* Value** | **Normal Distribution** |
| miR-144-3p | 34.90 | 30.86 | 38.08 | 1.68 | 0.0800 | Yes |
| miR-19a-3p | 31.13 | 26.57 | 35.30 | 1.72 | 0.0017 | No |
| miR-20a-5p | 31.33 | 27.67 | 35.70 | 1.67 | 0.0016 | No |
| miR-19b-3p | 30.28 | 25.96 | 33.83 | 1.64 | 0.0040 | No |
| miR-423-5p | 31.94 | 27.15 | 36.55 | 1.66 | 0.0990 | Yes |
| miR-23a-3p | 32.38 | 26.48 | 39.39 | 2.53 | 0.2620 | Yes |
| miR-24-3p | 30.78 | 27.03 | 35.33 | 1.63 | 0.0010 | No |
| miR-22-3p | 34.41 | 29.99 | 40.28 | 2.06 | 0.0006 | No |
| miR-122-5p | 33.95 | 28.92 | 44.66 | 3.63 | <0.0001 | No |
| miR-183-5p | 39.50 | 33.50 | 47.48 | 1.05 | <0.0001 | No |
| miR-5100 | 31.34 | 25.97 | 35.79 | 1.71 | 0.0432 | No |
| miR-4419a | 36.27 | 33.37 | 39.94 | 1.38 | 0.1247 | Yes |
| miR-125b-2-3p | 35.59 | 29.95 | 40.26 | 2.05 | 0.2065 | Yes |
| miR-150-3p | 34.29 | 31.14 | 35.91 | 1.05 | 0.7484 | Yes |
| miR-16-5p | 26.28 | 21.49 | 30.39 | 1.80 | 0.0510 | Yes |

CT, threshold cycle; SD, standard deviation.
